# Molecular detection of *Histoplasma capsulatum* in environmental samples collected from South African caves

**DOI:** 10.1371/journal.pntd.0013778

**Published:** 2025-12-22

**Authors:** Tsidiso G. Maphanga, Rutendo E. Mapengo, Oresti Ventouras, Serisha D. Naicker, Nelesh P. Govender

**Affiliations:** 1 Wits Mycology Division, School of Pathology, Faculty of Health Sciences, University of the Witwatersrand, Johannesburg, South Africa; 2 National Institute for Communicable Diseases, a Division of the National Health Laboratory Service, Centre for Healthcare-Associated Infections, Antimicrobial Resistance and Mycoses, Johannesburg, South Africa; 3 Subterranean Ecosystems Conservation Organization, Cape Town, South Africa; 4 Division of Medical Microbiology, Faculty of Health Sciences, University of Cape Town, Cape Town, South Africa; 5 Medical Research Council Centre for Medical Mycology, University of Exeter, Exeter, United Kingdom; Albert Einstein College of Medicine, UNITED STATES OF AMERICA

## Abstract

**Introduction:**

*Histoplasma capsulatum* naturally occurs in cave soil enriched by bat guano. South African caves are documented as probable sources of exposure for speleologists, casual visitors, or guano miners with several outbreaks of acute pulmonary histoplasmosis reported since 1977. Sporadic cases of disseminated histoplasmosis occur in South Africans living with advanced HIV disease. However, detection from the environment has not been confirmed. We used molecular assays to detect and confirm the presence of *H. capsulatum* in regularly-explored caves.

**Methods:**

Environmental samples were collected by a speleologist from seven South African caves from December 2020 to September 2021 in the Gauteng, Northern Cape and Western Cape provinces of South Africa and stored at 2–8 ^°^C. DNA was extracted directly from the samples using DNeasy PowerSoil Pro Kit. In-house internal transcribed spacer (*ITS*) panfungal polymerase chain reaction (PCR), pan-dimorphic reverse transcriptase-quantitative (RT-q) PCR and nested Hc100 PCR assays were used to detect *H. capsulatum*. Sequence identity was confirmed using the National Centre for Biotechnology Information (NCBI) BLAST tool following Sanger sequencing of the Hc100 nested-PCR product.

**Results:**

*H. capsulatum* was detected in five of the seven caves. Of 56 samples tested, 18 (32%) were positive from three caves in Gauteng Province [cave 1 (3/10); cave 2 (7/10); cave 3 (5/10)], one cave in the Western Cape Province [cave 4 (2/5)] and one cave in the Northern Cape Province [cave 6 (1/10)]. These samples were positive either by RT-qPCR or Hc100 PCR assays. Both RT-qPCR and Hc100 PCR assays were positive in 21% (12/56) samples. Seven percent (4/56) of samples were only RT-qPCR assay-positive and 4% (2/56) only Hc100 PCR-positive. Phylogenetic analysis of the Hc100 gene product from 10 samples (with good-quality sequences) identified four groups. Group 1 consisted of three samples from caves 1, 3, and 6 (Gauteng/ Northern Cape); Group 2 included four samples from caves 1, 2, and 3 (Gauteng); Group 3 had one sample from cave 4 (Western Cape); and Group 4 included two samples from caves 1 and 3 (Gauteng). None of the 56 samples tested positive with the *ITS* PCR assay.

**Conclusions:**

*H. capsulatum* is probably present in several regularly-explored caves with bat populations. This finding should be confirmed by culture. The RT-qPCR and the Hc100 PCR assays could be useful tools for wider environmental surveillance.

## Author summary

Simson and Barnetson reported the first documented case of disseminated histoplasmosis in South Africa in 1942. Cases of disseminated histoplasmosis have increasingly been reported in recent decades among people living with HIV in South Africa, making histoplasmosis the third most common laboratory-confirmed endemic mycosis. In addition, several outbreaks of pulmonary histoplasmosis have occurred among cave explorers, highlighting a possible link between cave environments and *Histoplasma* exposure, even though the fungus has not been successfully cultured directly from soil samples. From December 2020 to September 2021, we collected environmental samples from seven bat-inhabited caves across South Africa and tested 56 samples with three molecular assays. The RT-qPCR and Hc100 PCR assays detected *Histoplasma* nucleic acids from 18 samples in five caves. In contrast, the *ITS* PCR assay was negative on all samples. Cluster analysis further revealed that the environmental *Histoplasma* strains were closely related to clinical isolates from South African patients. These results reinforce the importance of taking protective measures such as wearing masks or respirators when entering caves, especially during activities that may disturb guano and release fungal conidia.

## Introduction

*Histoplasma capsulatum*, which is now known to comprise of several pathogenic cryptic species (including a distinct African lineage), causes human histoplasmosis in the Americas, Africa, Asia and Oceania [1–3]. In South Africa, a case of disseminated histoplasmosis was first recorded in 1942 but is now most often diagnosed among people living with HIV [4,5]. Outbreaks of acute pulmonary histoplasmosis have also been reported [1]. The mycelial phase of this thermally-dimorphic fungus is found in guano-enriched soil [6] and is known to be harboured in the soil around river basins [7–9]. The fungus thrives in areas with high nitrogen content, humidity of >60%, temperature of 18^°^C-28^°^C, darkness and naturally occurs in caves, woods, old buildings, and bird farms [10]. Acute human infection is linked to occupational exposure and recreational activities as a result of microconidia being released into the air when the soil is disturbed [11]. Once the conidia are inhaled, human body temperature stimulates a transition from a mycelial to yeast form [12]. Disease manifestations depend on host immunity and the quantity of inhaled conidia, ranging from an acute febrile illness to severe multi-system disseminated disease [13,14]. Testing soil could identify environmental hotspots that people with risk factors should avoid.

The first research on the natural habitat of *Histoplasma* spp. began in the 1940s and 1950s, with a focus on chicken houses [15,16]. Cave-associated histoplasmosis has been reported in several studies [16–18], including outbreaks across several regions of the world, including in South Africa [19–25]. These outbreaks showed an association between *H. capsulatum* and bird/ bat guano [26–28]. They also helped delineate populations at higher risk of acute infection such as speleologists, eco-tourists, archaeologists, construction workers, bird farmers, and organic fertilizer handlers [28,29]. South African caves in the Western Cape and Gauteng provinces were documented as probable sources of exposure for speleologists, casual visitors, or guano miners with several outbreaks of acute pulmonary histoplasmosis reported since 1957 among new members of speleological societies [6,25]. These outbreaks were never confirmed by laboratory testing of clinical or environmental samples. In general, it is typically challenging to isolate *Histoplasma* in culture directly from soil samples owing to a high load of fast-growing environmental fungi such as *Aspergillus* species. [7]. However, several studies have used molecular assays to detect *H. capsulatum* nucleic acids in soil from Brazil, Mexico, and the United States of America [7,30–33]. The Hc100 nested polymerase chain reaction (PCR) has a sensitivity of 54% and specificity of 100% in detecting *Histoplasma* in clinical samples compared to culture and serological tests [29,34]. An internal transcribed spacer (*ITS)* PCR assay has been used to screen for the phylogenetically closely-related fungus, *Emergomyces africanus* in soil samples in South Africa; 30% of the 60 samples were positive [35]. A pandimorphic reverse transcriptase-quantitative (RT-q) PCR assay has been previously used for screening of clinical samples and cross-reactions have been reported with several *Blastomyces* species [5]. Molecular assays used for screening of clinical samples for *Histoplasma* could be adapted to test environmental samples. We evaluated a conventional panfungal *ITS* PCR assay, conventional Hc100 nested PCR assay and a pandimorphic RT-qPCR assay to detect *H. capsulatum* directly from environmental samples collected from bat-inhabited caves in South Africa.

## Materials and methods

### Environmental sample collection

**Fig 1:**
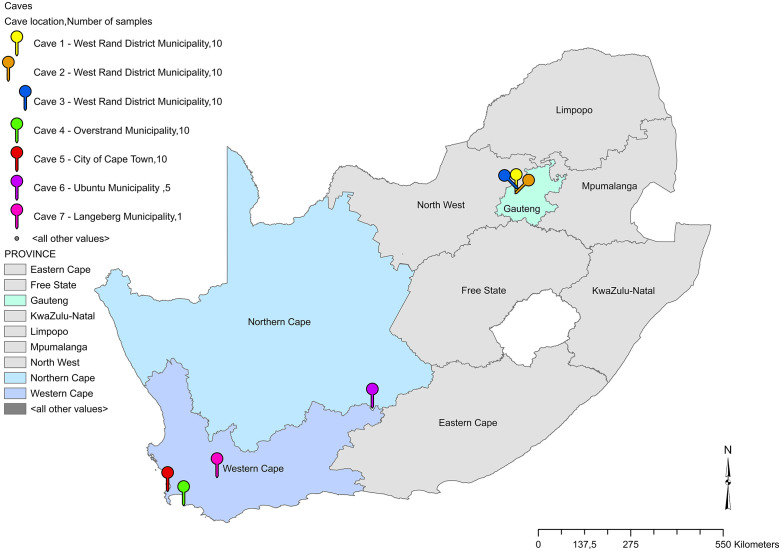
Map showing the approximate location of the caves in the three South African provinces where the environmental samples were collected. Gauteng Province: cave 1 to cave 3 (West Rand District Municipality), Western Cape Province: cave 4 (Overstrand Local Municipality), cave 5 (City of Cape Town), cave 7 (Langeberg Municipality), Northern Cape: cave 6 (Ubuntu Local Municipality). ArcGIS Pro was used to map the provinces were the cave samples were collected. Shapefiles (sa_dc20090310.shp) were sourced from the open-source ArcGIS platform. The provincial boundaries were obtained from Statistics South Africa (Stats SA).https://www.statssa.gov.za/?page_id=964. Used Esri Developer resources. According to Esri’s guidelines, the appropriate attribution is: Sources: Statistics South Africa Powered by Esri.

Fifty-six environmental samples were collected from seven South African bat-inhabited caves from December 2020 through to September 2021. The exact locations of the caves are not shared, but the sampling sites were situated in named municipalities within the Gauteng, Western Cape Province and Northern Cape provinces. Three caves are located in the Western Cape Province (Overstrand, City of Cape Town and Langeberg Municipalities), three in Gauteng Province (West Rand Municipality) and one in the Northern Cape Province (Ubuntu Municipality). All caves were sampled by convenience as speleologists were exploring these caves (Fig 1). Ten soil or guano samples were collected per cave in the three caves in Gauteng and two caves in Western Cape. Five samples were collected in the Northern Cape cave and a single sample collected from the remaining Western Cape cave. The environmental samples were labelled by the collectors as guano (n = 21), soil with bat guano (n = 16), soil (n = 13), fungus/ guano (n = 5) and owl guano (n = 1).

We assume that *H. capsulatum* may be present in these caves based on previous reports. In South Africa, 133 cases of histoplasmosis (46 proven, 67 probable, 20 possible) were reported between 2010 and 2020 [5]. Several of these cases were diagnosed in hospitals located in the same municipalities as caves sampled in this study: 10 cases in the West Rand Municipality, Gauteng Province (caves 1–3), 18 cases in the City of Cape Town Metro, Western Cape Province (cave 4), and five cases each in the Overstrand and Langeberg municipalities, Western Cape Province (caves 5 and 7). Furthermore, other reports have documented possible cases of histoplasmosis among cavers in both the Western Cape and Gauteng provinces [6,25].

### Handling and processing of environmental samples

The samples were sent to the National Institute for Communicable Diseases (NICD) following International Carriage of Dangerous Goods by Road (ADR) safety regulations and the UN3373 Biological substance Category B packaging requirements [36]. Upon arrival, the samples were stored at 4°C to 8°C until processed. Handling and processing of the samples was conducted in a biosafety level 3 facility with personnel wearing appropriate personal protective equipment including a fit-tested N95 mask and gloves, as the environmental samples may pose a potential risk of infection.

### In-house validation of molecular PCR assays

A total of 16 soil samples collected from different points around the National Institute for Communicable Diseases (NICD) campus in Johannesburg were used during validation of the three molecular assays (*ITS* panfungal PCR, Hc100 nested PCR and pandimorphic RT-qPCR). The soil samples were assumed to harbour environmental fungi other than *H. capsulatum.* These soil samples were used as negative controls to determine the specificity of the assays. One soil sample was randomly selected from the 16 to determine the limit of detection (LOD). A portion of the same soil sample was autoclaved. The autoclaved and non-autoclaved soil samples were spiked with 1.5X10^3^ CFU of *H. capsulatum* mould and thereafter, serial dilutions (1:10, 1:100, 1:1000) were made.

We also validated the assays using DNA extracted from cultured fungi and stored in the -20°C freezer at NICD. Species identity from the DNA extracts was previously confirmed using Sanger sequencing of the *ITS*, calmodulin or beta-tubulin genes. DNA of *H. capsulatum* (n = 14), and *H. capsulatum* var. *farciminosum* (*H. farciminosum*) (n = 2) were used to determine the sensitivity of the assay. The LOD was determined using 1:10 serial dilutions of 0.1 ng/µl *H. capsulatum* for the RT-qPCR assay and 10 ng/µL for the Hc100 and *ITS* PCR assays. The specificity was also determined using 63 fungal pathogens: 25 moulds, 18 yeasts, 16 closely-related thermally-dimorphic fungi [*E. africanus* (n = 10), *E. pasteurianus* (n = 1), *Blastomyces dermatitidis* (n = 1), *B. emzantsi* (n = 2), *B. percursus* (n = 2)] and 4 non-related thermally-dimorphic fungi [*S. schenckii* (n = 4].

### DNA extraction and conventional PCR assays from environmental samples

Genomic DNA was extracted from the environmental samples using a DNeasy PowerSoil Pro Kit (Qiagen, Hilden, Germany) as per the manufacturer’s instructions. PCR was performed immediately after nucleic acid extraction. We used *ITS* 1 and 4 primers to amplify the DNA from the environmental samples as previously described by White et al., 1990 [37]. The Hc100 PCR was performed using two sets of primers according to Gómez et al. 2018 with minor modifications [29]. The Hc100 PCR method included the use of two sets of primers designed by Bialek in 2001 [33]. We used these primers to amplify the DNA from the environmental samples and clinical samples. A total volume of 50 µl consisting of 2.5 µl buffer (10X), 0.75 µl MgCl_2_ (25mM), 1 µl deoxynucleoside triphosphate (10 mM), 0.2µl Hotstart DNA polymerase (5 U/ul) (Thermo Fisher Scientific, South Africa), 38.55 µl nucleic free water, 1 µl each forward and reverse primer (10 uM) (Inqaba Biotechnical Industries (Pty) Ltd, South Africa) and 5 µl of DNA from environmental samples/2 µl of DNA from clinical and horse isolates. The amplified product was diluted (1:10) and the 2^nd^ PCR consisted of 0.83 µl buffer (10X), 0.25 µl MgCl_2_ (25mM), 0.3 µl deoxynucleoside triphosphate (10 mM), 0.06 µl Hotstart DNA polymerase (5 U/ul) (Thermo Fisher Scientific, South Africa), 21.26 µl nucleic free water, 0.3 µl each forward and reverse primer (10 uM) (Inqaba Biotechnical Industries (Pty) Ltd, South Africa) and 1.7 µl of diluted DNA in a final volume of 25 µl. The thermal cycler conditions for both PCRs were set as described by Gómez et al. 2018 [29].

The *ITS* and nested Hc100 products were sequenced by Sanger sequencing on a 3130 sequencer (Applied Biosystems, Life Technologies Corporation, USA). Sequences were viewed and manually edited using Chromas lite. Species identification was performed using the National Center for Biotechnology Information (NCBI), nucleotide Basic Local Alignment Search Tool (BLAST) database (https://blast.ncbi.nlm.nih.gov/Blast.cgi).

### Cluster identification

The sequences from the nested Hc100 PCR were used to determine phylogenetic relatedness of the environmental *H. capsulatum* sequences. We also included 14 *H. capsulatum* sequences obtained from South African clinical isolates and the two *H. farciminosum* sequences from horse isolates. A consensus from the forward and reverse sequences of Hc100 gene was generated and alignment was performed using BioEdit version 7. The phylogenetic tree was constructed in MEGA version 11 by a maximum likelihood statistical method using 1000 bootstrap replicates. Sequences were deposited into NCBI database with accession numbers PV953867-PV953868, PV975993-PV976013.

### Pandimorphic PCR assay

A pandimorphic RT-qPCR assay targeting the mitochondrial small subunit (mtSSU) of *E. africanus* or *H. capsulatum* was used for the detection of *H. capsulatum* directly from environmental samples. PCR was performed according to Mapengo et al., 2022 [5]. A cycle threshold (Ct) value of ≤40 was considered positive, while any Ct above 40 was considered negative. This assay cross-reacts with *B. dermatitidis, B. percursus* and *B. emzantsi* [5].

### Quality control

*Candida albicans* ATCC 90028 was used as a positive control for the *ITS* panfungal PCR assay. *H. capsulatum* clinical NICD-MRL_603 was used as a positive control for the Hc100 PCR, while *H. capsulatum* clinical NICD-MRL_603 and NCPF4091 *E. africanus* were included as positive controls for the pandimorphic RT-qPCR assay. We included nuclease-free water as a negative control. Internal controls, such as Lambda DNA and RNA virus, were utilized during Hc100 and pan-dimorphic PCRs, respectively. These controls helped to monitor potential PCR failures that could have arisen from the presence of inhibitors in the environmental samples.

### Statistical analysis

The sensitivity, specificity, positivity and negativity rate of the molecular methods in detecting *H. capsulatum* in the environmental samples were calculated using Stata version 14.0 (StataCorp, USA).

## Results

### In-house validation of molecular PCR assays

#### NICD soil samples.

All 16 soil samples of various types collected around the NICD campus in Sandringham, Johannesburg were negative for *H. capsulatum* with the pandimorphic RT-qPCR, Hc100 nested PCR and *ITS* PCR. Environmental fungi such as *Epicoccum nigrum, Macrophoma lageniformis, Lophiostoma corticola, Stagonospora cirsii, Pleosporales, Leptosphaerulina, Ascobolus*, *Epicoccum italicum, Diymella s*pecies*, Phaeomoniella chlamydospora* and *Scleroderma capeverdeanum* were identified with the *ITS* PCR assay following Sanger sequencing. No PCR inhibition was observed; the Lambda DNA and RNA virus were positive during Hc100 nested PCR and pan-dimorphic PCR runs respectively. The LOD determined using 1:10 serial dilutions of soil spiked with *H. capsulatum* mould was as follows: pandimorphic RT-qPCR (autoclaved: 1.5X10^-2^ cells/ml [0.003 cells/ml] and non-autoclaved soil: 1.5 X 10^1^ [30 cells/ml]); Hc100 nested PCR (autoclaved and non-autoclaved soil: 1.5 X 10^1^ [30 cells/ml]; *ITS* panfungal PCR (only detected in autoclaved soil: 1.5 X 10^2^ [300 cells/ml])*.*

#### DNA from cultured isolates.

The *ITS* PCR, Hc100 nested PCR and pandimorphic RT-qPCR all had a sensitivity of 100%, while the specificity was 100%, 97% and 90%, respectively (Table 1). Cross-reaction was noted with *H. farciminosum* DNA and an identity of *H. capsulatum* was obtained following sequencing of the Hc100 nested amplicon with query cover of 98% and similarity score of 96%. No cross-reaction was observed for the Hc100 nested PCR with DNA from any fungus including closely-related fungi such as *E. africanus, E. pasteurianus, B. dermatitidis, B. percursus* and *B. emzantsi.* Cross-reaction was observed for pandimorphic RT-PCR assay with DNA from *Blastomyces dermititidis*, *Blastomyces percursus* and *Blastomyces emzantsi*. The limit of detection obtained using DNA extracted from *Histoplasma* cultured isolates was as follows: pan-dimorphic RT-qPCR (DNA: 10^-9^ ng/µl [0.000000001 ng/µl], Hc100 nested PCR (DNA: 10^-4^ ng/µl [0.0001 ng/µl) and *ITS* panfungal PCR (DNA: 10^-3^ ng/µl [0.001 ng/µl).

**Table 1:** Sensitivity and specificity for ITS, Hc100 nested PCR and pandimorphic RT-qPCR assays using DNA from 79 cultured isolates.

	Conventional panfungal *ITS* PCR assay	Conventional Hc100 nested-PCR assay	Pandimorphic RT-qPCR assay
	Target: internal transcribed spacer gene	Target: gene encoding 100 kDa protein	Target: mitochondrial small subunit gene
Sensitivity n (%)	79 (100)	79 (100)	79 (100)
Specificity n (%)	79 (100)	77 (97)	74 (90)
**Total tested**	**79**	**79**	**79**

### Environmental samples from South African caves

*H. capsulatum* was detected in 5 of the 7 caves with the molecular assays. These included three caves (caves 1, 2 and 3) in Gauteng Province, one cave in the Western Cape Province (cave 4) and one cave in the Northern Cape Province (cave 6) (Table 2). The approximate locations of the sampled caves are shown in the map (Fig 1).

**Table 2:** Number of cave samples positive for Histoplasma capsulatum with three molecular assays.

Collection site	Collection date	No. of environmental samples	Pan-dimorphic RT-qPCR assay positive	Hc100 Fungal PCR assay positive	*ITS* panfungal PCR assay positive	Documented presence of *H. capsulatum*
Cave 1 (GP)	19-08-2021	10	3	3	0	Yes
Cave 2 (GP)	17-09-2021	10	7	4	0	Yes
Cave 3 (GP)	22-09-2021	10	3	5	0	Yes
Cave 4 (WC)	24-04-2021	10	2	1	0	Yes
Cave 5 (WC)	05-12-2020	10	0	0	0	No
Cave 6 (NC)	28-03-2021	5	1	1	0	Yes
Cave 7 (WC)	16-04-2021	1	0	0	0	No
Total		56	16	14	0	

Of 56 samples tested, 18 (32%) were positive from three caves in Gauteng [cave 1 (3/10); cave 2 (7/10); cave 3 (5/10)], one cave in the Western Cape [cave 4 (2/10) and one in the Northern Cape [cave 6 (1/5)] (Table 2). These samples were positive either by RT-qPCR or Hc100 PCR assays. Both RT-qPCR and Hc100 PCR assays were positive in 21% (12/56) samples. Seven percent (4/56) of samples were only RT-qPCR assay-positive and 4% (2/56) were only Hc100 PCR-positive (Table 2).

The 14 environmental samples that were positive with the Hc100 PCR assay had an identity of *H. capsulatum* with a similarity score of ≥90% based on pairwise alignment on BLAST. For the RT-qPCR assay, the 16 environmental samples that were positive had a median cycle threshold value of 35 with a range spanning from 26 to 40. None of the 56 sampleswere positive for *H. capsulatum* with the *ITS* panfungal PCR assay. However, 55% (31/56) of the samples were negative for fungi while 45% (25/56) were positive for other fungi found in environment; these included *Gamsia* species (n = 6), *Chrysosporium* species (n = 3), *Cladosporium* species (n = 2), *Ascogregarina* species (n = 2), *Fusarium* species (n = 1), *Penicillium* species (n = 1), *Talaromyces* species (n = 2), *Mucor* species (n = 1), *Diploschiste* species (n = 1), *Parengyodontium album* (n = 1), *Cutaneotrichosporon* species (n = 1), *Arthroderma uncinatum* (n = 1), *Aspergillus versicolor* (n = 1), *Alternaria* species (n = 1) and uncultured *Trichosporonales* (n = 1) (Table 3). Of the 56 environmental samples, *Histoplasma* was detected in 4 guano samples, 3 soil with guano samples, 5 soil samples and 3 fungus/ guano samples. The owl guano sample was negative for *Histoplasma*.

**Table 3:** Molecular results for the environmental samples collected from South African caves (2020 to 2021).

Cave	Collection Date	Sample #	Material	Pandimorphic RT-qPCR	Hc100 PCR	*ITS* PCR
**Cave 1 (Gauteng)**	**19-08-2021**	1	Owl guano	Negative	Negative	Negative
		2	Soil	Positive	Positive	*Alternaria* species
		3	Guano	Negative	Negative	*Cladosporium/Penicillum* species
		4	Soil	Positive	Positive	*Fusarium* species
		5	Soil	Negative	Negative	Negative
		6	Soil	Negative	Negative	Negative
		7	Soil	Positive	Positive	Negative
		8	Soil	Negative	Negative	*Aspergillus versicolor*
		9	Guano	Negative	Negative	*Ascogregarina* species
		10	Guano	Negative	Negative	*Chrysosporium pallidum*
**Cave 2 (Gauteng)**	**17-09-2021**	1	Guano	Negative	Negative	Negative
		2	Guano + fungus	Positive	Positive	*Gamsia* species/ *Chrysosporium carmichaelii*
		3	Guano	Positive	Negative	*Talaromyces* species
		4	Guano	Positive	Positive	Negative
		5	Soil	Positive	Positive	*Penicillium crustosum*
		6	Guano	Negative	Negative	*Ascogregarina culicis*
		7	Guano + fungus	Negative	Negative	*Gamsia* species
		8	Guano	Positive	Negative	*Arthroderma uncinatum/ Trichophyton simii*
		9	Guano	Positive	Negative	Negative
		10	Guano + fungus	Positive	Positive	*Chrysosporium carmichaelii*
**Cave 3** **(Gauteng)**	**22-**09**-2021**	1	Soil	Negative	Negative	Negative
		2	Guano + fungus	Negative	Negative	*Ascogregarina culicis*
		3	Guano + fungus	Positive	Positive	Negative
		4	Soil	Negative	Positive	Negative
		5	Soil	Negative	Negative	Negative
		6	Guano	Negative	Positive	*Gamsia* species
		7	Soil	Negative	Negative	Uncultured Trichosporonales
		8	Soil	Positive	Positive	Negative
		9	Soil	Negative	Negative	Negative
		10	Guano	Positive	Positive	*Gamsia kooimaniorum*
**Cave 4 (Western Cape)**	**24-04-2021**	1	Guano + Soil	Positive	Positive	*Mucor flavus*
		2	Guano + Soil	Negative	Negative	Negative
		3	Guano + Soil	Negative	Negative	*Gamsia* species
		4	Guano + Soil	Negative	Negative	*Cutaneotrichosporon*
		5	Guano + Soil	Negative	Negative	Negative
		6	Guano + Soil	Negative	Negative	*Diploschiste scruposus*
		7	Guano + Soil	Negative	Negative	Negative
		8	Guano + Soil	Negative	Negative	Negative
		9	Guano + Soil	Positive	Negative	Negative
		10	Guano + Soil	Negative	Negative	Negative
**Cave 5 (Western Cape)**	**5-12-2020**	1	Guano	Negative	Negative	Negative
		2	Guano	Negative	Negative	Negative
		3	Guano	Negative	Negative	*Gamsia* species
		4	Guano	Negative	Negative	Negative
		5	Guano	Negative	Negative	Negative
		6	Guano	Negative	Negative	Negative
		7	Guano	Negative	Negative	Negative
		8	Guano	Negative	Negative	*Cladosporium cladosporioides*
		9	Guano	Negative	Negative	Negative
		10	Guano	Negative	Negative	Negative
**Cave 6 (Northern Cape)**	**28-03-2021**	1	Guano + Soil	Negative	Negative	Negative
		2	Guano + Soil	Negative	Negative	Negative
		3	Guano + Soil	Negative	Negative	*Talaromyces* species
		4	Guano + Soil	Positive	Positive	*Parengyodontium album*
		5	Guano + Soil	Negative	Negative	Negative
**Cave 7 (Western Cape)**	**16-04-2021**	1	Guano + Soil	Negative	Negative	Negative

### Phylogenetic analysis of Hc100 PCR product sequences

Ten of the 14 environmental samples that were positive for *H. capsulatum* with the Hc100 nested PCR were included in the phylogenetic analysis. Poor sequences were obtained from the remaining four samples. These environmental samples were collected from three caves in Gauteng Provinces (cave 1: 3/3; cave 2: 1/4; cave 3: 4/5), one cave in the Western Cape (cave 4: 1/1) and one cave in the Northern Cape (cave 6: 1/1) (Table 2).

**Fig 2:**
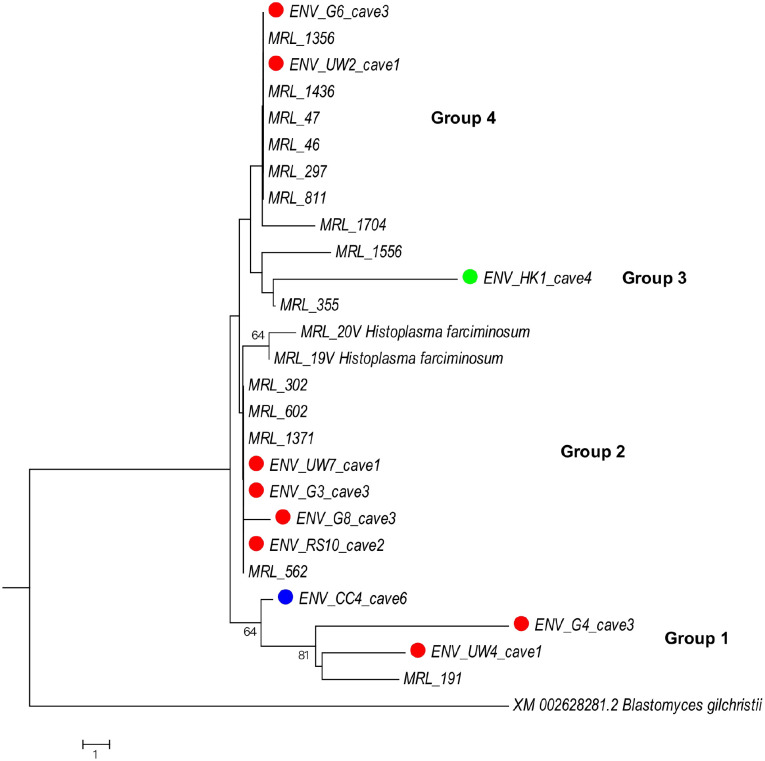
The phylogenetic tree is based on the Hc100 sequences showing diversity of ten *Histoplasma* from South African caves samples [red: samples from Gauteng province (n = 8); blue: Northern Cape province (n = 1) and green: Western Cape province (n = 1)]. We included 14 South African clinical *Histoplasma capsulatum* isolates (named MRL_1356, MRL_1436, MRL_475, MRL_460, MRL_297, MRL_1704, MRL_1556, MRL_355, MRL_191, MRL_562, MRL_302, MRL_602, MRL_1371, MRL_811) and two horse *Histoplasma* var *farciminosum* isolates (MRL_20V and MRL_19V). The phylogenetic tree was generated using a maximum likelihood algorithm with 1000 bootstrap replications, following the Kimura 2 parameter model. *Blastomyces dermatitidis/ gilchristii* XM002628281 strain was used as an out-group.

Phylogenetic analysis revealed four distinct groups. Group 1 included three environmental samples, two from Gauteng (cave 1: n = 1; cave 3: n = 1) and one from Northern Cape (cave 6: n = 1) which clustered with one South African clinical *H. capsulatum* sequence. Group 2 comprised four environmental samples from Gauteng (cave 1: n = 1; cave 2: n = 1; cave 3: n = 2), which clustered with four South African clinical *H. capsulatum* sequences. Group 3 consisted of one environmental sample from Western Cape Province (cave 4), clustering with two South African clinical *H. capsulatum* sequences. Group 4 included two environmental samples from Gauteng Province (cave 1: n = 1 and cave 3: n = 1), which also clustered with seven South African clinical *H. capsulatum* sequences (Fig 2). The two horse sequences of *H. farciminosum* did not cluster with any South African clinical *H. capsulatum* sequences.

## Discussion

We used three different molecular methods to screen for *H. capsulatum* nucleic acids in environmental samples; *H. capsulatum* was detected in five of the seven South African bat-inhabited caves by two of these molecular assays. Molecular methods can rapidly detect *H. capsulatum* from clinical or environmental samples. These assays have a better yield in soil than culture because *H. capsulatum* takes a long time to grow and is frequently overgrown by fast-growing environmental fungi such as *Aspergillus*.

The test positivity in soil was 29% with the pandimorphic RT-qPCR assay and 25% with the Hc100 nested PCR assay. Twelve samples were positive with both the pan-dimorphic PCR and Hc100 nested PCR, indicating a high degree of concordance (12/18; 67%) for both tests and giving more confidence in the results. None of the environmental samples were positive with the *ITS* PCR assay. No major difference was noted between the pandimorphic RT-qPCR and the Hc100 nested PCR assay in detecting *H. capsulatum* from the environmental cave samples but from the validation results, the RT-qPCR assay had a lower LOD. RT-qPCR assays are generally more sensitive than conventional PCR assays since they can detect a small amount of target DNA in samples. In our study, the RT-qPCR assay’s LOD was 10^-9^ ng/µl and the Hc100 nested PCR’s LOD was 10^-4^ ng/µl. The pandimorphic RT-qPCR assay could be more useful in detecting *H. capsulatum* from environmental samples with a shorter turnaround time versus the Hc100 nested PCR which requires the performance of two different PCR assays plus sequencing. We do not recommend the use of the *ITS* panfungal conventional PCR in testing environmental samples because it is not specific for *H. capsulatum* and the dominant fungi in environmental samples are probably preferentially amplified with the assay.

For the nested Hc100 PCR assay, an NCBI BLAST search confirmed the presence of the *H. capsulatum* 100kDa region in the environmental samples. Phylogenetic analysis of the environmental samples revealed four distinct groups, each clustering with sequences from known South African clinical strains of *H. capsulatum*. The geographic clustering of these environmental sequences into different phylogenetic groups suggests a high level of strain diversity in the environment. A recent genomic epidemiological study that included South African clinical isolates also demonstrated diversity of *H. capsulatum* in Africa, identifying more than five distinct groups [3]. In this study, the environmental samples clustered within the four previously recognized groups, indicating that no new genotypes were detected. Nevertheless, other studies have reported the emergence of new genotypes in Brazil, particularly in the northeastern region and the state of Ceará [38,39]. The emergence of these new genotypes in *Histoplasma* is likely driven by selective pressure, prompting rapid adaptation to changes in environmental conditions [10]. Hence, it would be important to conduct environmental surveillance of these pathogens in order to monitor the emergence of new genotypes across Africa.

Although the two *H. farciminosum* isolates were identified as *H. capsulatum* on NCBI BLAST, they did not cluster with any of the environmental and South African *H. capsulatum* strains, suggesting they are a distinct species. To date, the nested Hc100 PCR assay has been validated using *H. capsulatum* and *Histoplasma. var. duboisii* (*H. duboisii*) DNA but not *H. farciminosum* DNA. This could explain the misidentification of *H. farciminosum* as *H. capsulatum*, as Hc100 sequences for *H. farciminosum* may not be available on NCBI BLAST. That being said, a nested PCR targeting the *ITS* region of the rRNA operon has previously been evaluated and recommended for screening *H. farciminosum* in equines [40].

All PCR assays were negative in two of the seven caves tested. A negative PCR in these caves does not exclude *Histoplasma* contamination. There is still a possibility that these cave samples were contaminated by bacteria and other fungi limiting the detection of *H. capsulatum*. It is important that proper measures still be put in place to avoid cave visitors coming in contact with the contaminated soil.

Outbreaks can be detected and controlled with the use of molecular tests that have shorter turnaround times. The Hc100 nested PCR was previously used to trace the source of infection for an outbreak that occurred in a hotel in Acapulco, Guerrero, Mexico leading to control measures to prevent more outbreaks in this hotel [19]. Our results showed that 25% of the environmental samples collected from South African caves were contaminated with *H. capsulatum*; this is higher than the 10.5% recorded from testing 239 environmental samples including composted organic fertiliser, chicken manure and soil samples from cave floors in Columbia (tested using the Hc100 nested PCR) [29]. A previous study by Gomez *et al* in 2019 also documented testing of 393 Colombian environmental samples using the nested Hc100 PCR with a positivity rate of 9.9%; this supports the need for more sampling as our study only had 56 environmental samples [41]. A more sensitive assay, the *Histoplasma* 100-kDa real-time PCR, was optimised for testing environmental samples by Gomez and others in 2022 [34]. They tested 332 samples collected in Columbia and detected *Histoplasma* in 80% of raw organic materials and 62% of composted materials; this supports optimising new molecular methods with shorter turnaround times when testing environmental samples [34].

Our results confirm the presence of *H. capsulatum* in several caves frequently visited by cave explorers. This information will alert speleologists to exercise caution and wear protective equipment when visiting the tested caves to prevent potential infections and will guide the relevant parks authorities in implementing guidance to members of the public to limit exposure and infections. A limitation of our study was sampling bias introduced by collecting samples only in certain bat-inhabited caves based on the assumption that *H. capsulatum* might be present with previously-reported cases of possible histoplasmosis [6,25,42,43]. Other studies have suggested that cave explorers might have been infected during their visits to these particular caves [25]. For example, a case of histoplasmosis was described in the second known outbreak in the old Cape Province (comprising the new Western, Eastern and Northern Cape provinces) and local cave explorers reported a suspicion of histoplasmosis contracted after visiting the caves [25]. The PCR-positive tests from our study should be interpreted with caution as the amount of viable fungus was not quantified; therefore, this is not equivalent to the infectivity and viability of the *H. capsulatum* present in these caves. We also do not have epidemiological data on actual infections that have occurred when people visited these caves. There is a need to improve the reporting of histoplasmosis infections among cave explorers. We did not compare molecular to culture-based methods in soil samples which typically involves passaging of soil samples through mice. Recovery of *H. capsulatum* using a mouse model presents challenges due to the need for animal facilities, time and expertise but this would be a valuable exercise.

In summary, environmental samples from five of seven South African bat-inhabited caves were PCR-positive for *H. capsulatum* by two different methods. Negative PCR results do not exclude *Histoplasma* contamination in the other two caves. There is a need for more environmental sampling across endemic areas in Africa including cave and non-cave samples for a better understanding of areas of possible *H. capsulatum* infection and presence of different genotypes. Appropriate measures such as wearing masks or respirators should be taken when exploring caves known to be contaminated with *H. capsulatum* to avoid exposure and subsequent infection with the fungus, particularly when handling samples contaminated with guano.
